# Supplementary material for: Upregulation of miR-33 Exacerbates Heat-Stress-Induced Apoptosis in Granulosa Cell and Follicular Atresia of Nile Tilapia (Oreochromis niloticus) by Targeting TGFβ1I1
Source: Genes (Basel). 2022 Jun 2;13(6):1009. doi: 10.3390/genes13061009 (PMC9222912; doi:10.3390/genes13061009)
Supplement: Supplementary file 1 [file genes-13-01009-s001.zip › Figures S1¿CS4.pdf]

A GenBank: XP_005463932.1

MEDLDALLADLESTSSPLPRCPVLLTSDPPQNSDPTTQDSAQTRPPPPAYTPQQ
TVSSAMKTTQNSTPDKLYSTVCKPRSPRSADPPLAFSSSSLLGGGLSELDHLLQ
ELNATQFNITDEILAQFPSSKKDERDNIKDKAPTSSSSSAKPSATSATLELDKLM
ASLSDFRVQSTPAAPVTPAPVTASPQQPAAAPPQPPSSGGSLDSMLGLLQSDLS
RQGVQTSSKGNCSACQKPVVGQVVTALGKVWHPEHFVCTECETELGSRNFF
EKDGRPYCEPDYFTLFSPHCAHCNKPILNKMVTALDKNWHPECFCCVKCSRA
FGEEGFHDREGQQYCQQCFLTLFASRCQGCSQPILENYISALNSLWHPQCFVC
RECYSPFVNGSFFEHEGKPLCEAHYHQSRGSMCQACQQPILGRCVTAMGAKF
HPHHLVCHFCLKPLSKGCFKEQENKPYCHPCFIKLFG

TGFβ1I1 protein sequence: QNSDPTTQDSAQTRP

B

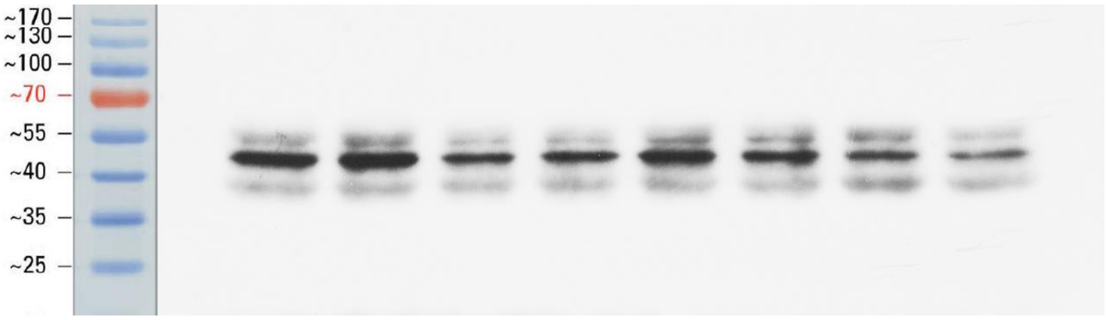

Figure S1 TGFβ1I1 protein sequence information and marker map of Nile tilapia *Oreochromis niloticus*.

A GenBank: AHN82370.1

IAALVVDNGSGMCKAGFAGDDAPRAVFPSIVGRPRHQGVMVGMGQKDSYV
GDEAQSKRGILTLKYPIEHGIVTNWDDMEKIWHHTFYNELRVAPEEHPVLLTE
APLNPKANREKMTQIMFETFNTPAMYVAIQAVLSLYASGRTTGIVMDSGDGV
THTVPIYEGYALPHAILRLDLAGRDLTDYLMKILTERGYSFTTTAEREIVRDIK
EKLCYVALDFEQEMGTAASSSSLEKSYELPDGQVITIGNERFRCPEALSQPSFL
GMESCGIHETTYNSIMKCDVDIRKDLYANTVLSGGTTMYPGIADRMQKEITA
LAPSTMKIKIIAPPERKYSVWIGGSILASLSTFQQMWISKQEYDESGPSIVHRKC
F

***β*-actin protein sequence:** ISKQEYDESGPSIV

B

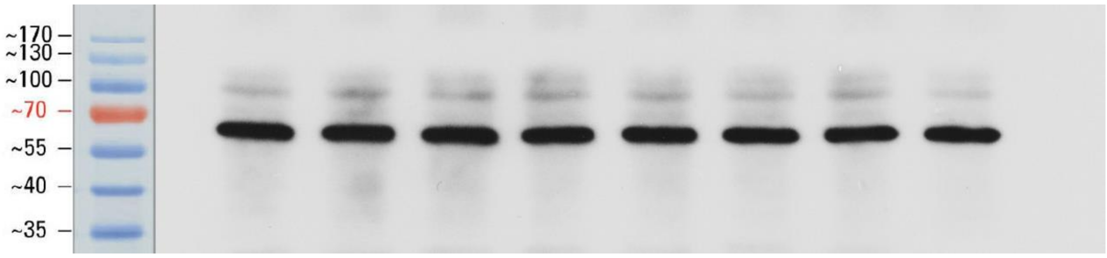

Figure S2 *β*-actin protein sequence information and marker map of Nile tilapia *Oreochromis niloticus*.

A

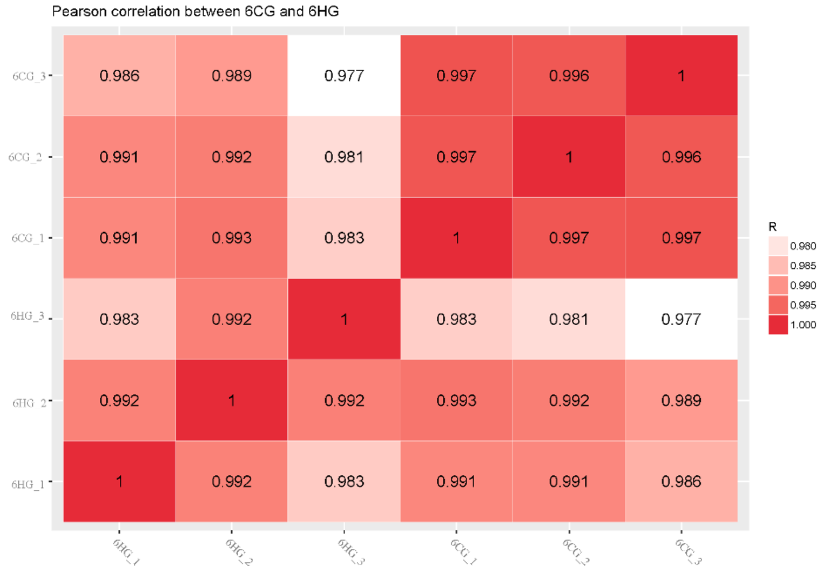

B

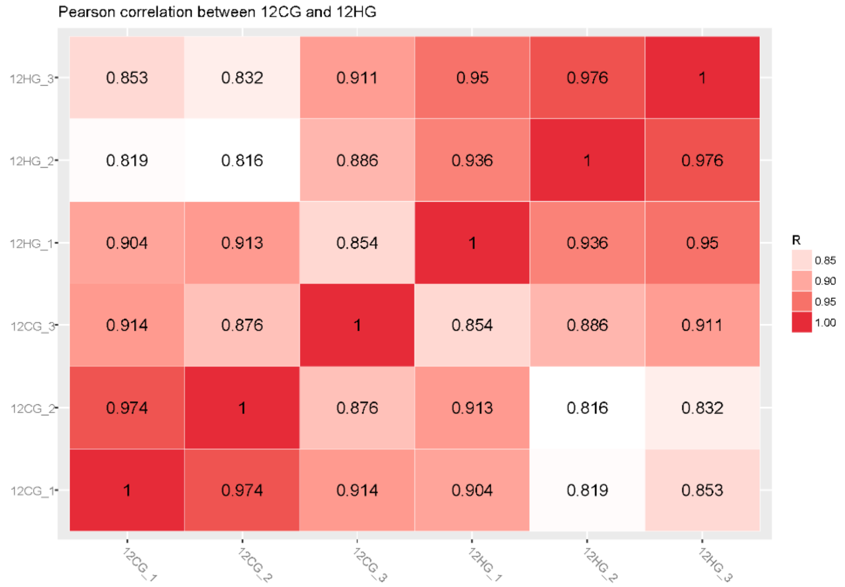

Figure S3 Quality assessment of miRNA sequencing results in 6CGs vs 6HGs and 12CGs vs 12HGs

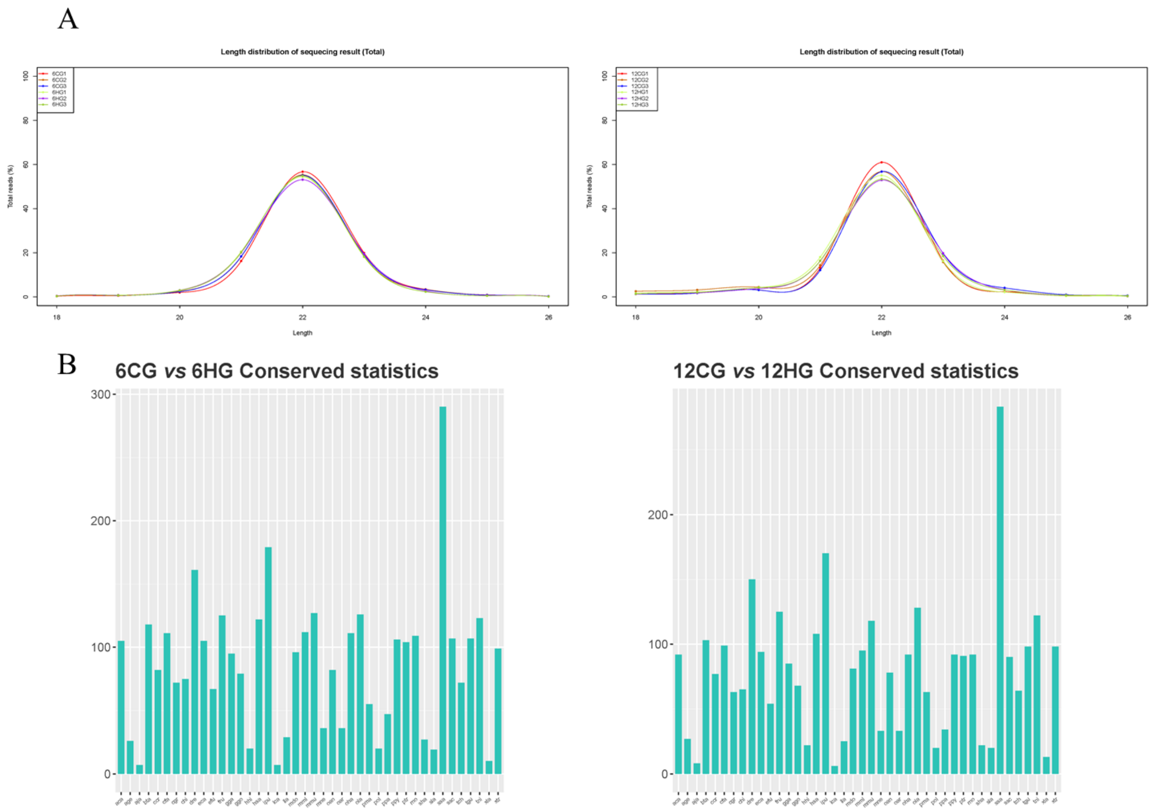

Figure S4 Length distribution of counts of total and unique sRNAs (A) and conservation profile of the identified miRNAs (B)
