# Supplementary material for: Upregulation of miR-33 Exacerbates Heat-Stress-Induced Apoptosis in Granulosa Cell and Follicular Atresia of Nile Tilapia (Oreochromis niloticus) by Targeting TGFβ1I1
Source: Genes (Basel). 2022 Jun 2;13(6):1009. doi: 10.3390/genes13061009 (PMC9222912; doi:10.3390/genes13061009)
Supplement: Supplementary file 1 [file genes-13-01009-s001.zip › Table S1.pdf]

Table S1 The primer sequence of the gene in this experiment

Name	Primer sequence (5'-3')
**Bax**	F:5’-ATTCGCTCCTACTTCGGCAC-3’<br>R:5’-GTTCTTCCTGTTGAGCGGGT-3’
**Caspase-3**	F:5’- TGAATTTCCGGGCCTGAGTG-3’<br>R:5’-GACAGACCGTCATCGTGCTT-3’
**Bcl-2**	F:5’-GACGATGATGCCAGGGAGAG-3’<br>R:5’- CTCAGAGTTCACTGGAGCGG-3’
**Caspase-8**	F:5’- CAGACTCAGCATGTCTGCCA-3’<br>R:5’- ATAACGTGTCCCTCCGCATC-3’
**TGFβ1I1**	F:5’- CAGGACCGACGCAGACAATA-3’<br>R:5’- CTCAGGCACTCCCAAATCCT-3’
**VEGFA**	F: 5’- CCAGTGAGAGCATACCTGGC -3’<br>R: 5’- TTTCCCCTCTTGGACACAGC -3’
**VEGFB**	F: 5’- CTGACGAGGCTTTCGAGTGT -3’<br>R: 5’- GTGTAGGCTGGAGGTGTTCC -3’
**KDR**	F: 5’- TCCACTGCAGGCTGTTTTCA -3’<br>R: 5’- CGCTGCAGCAAAACAAGTGA -3’
***β*-actin**	F:5’- CCACACAGTGCCCATCTACGA-3’<br>R:5’- CCACGCTCTGTCAGGATCTTCA -3’
**miR-27e**	AAGCGACCTCACACAGGTTGGC
**miR-27b-3p**	AACACGCACAGAATCGTTTCTG
**miR-33**	AACACGCAGCCACTGACTAAC
**miR-34a**	AAGCGACCTGTGTGTGTGTGTG
**miR-133a-5p**	AAGCGACCCTATACAGTCTACTG
**miR-301b-5p**	AACACGCCTGCCCTGGCCCGAG
